# Supplementary material for: Protocol for a qualiquantitative study of accessibility of sexual and reproductive health services among women with motor disabilities in Morocco
Source: PLoS One. 2024 Oct 29;19(10):e0311608. doi: 10.1371/journal.pone.0311608 (PMC11521270; doi:10.1371/journal.pone.0311608)
Supplement: S1 Table — ADL and IADL Domains. (DOCX) [file pone.0311608.s001.docx]

**Table 1 : disability staging system based on Activities of Daily Living (ADLs) and Instrumental Activities of Daily Living (IADLs)**

| **Stage** | **ADL Domain** | **IADL Domain** |
| --- | --- | --- |
| Stage 0 : no disability | Can eat, toilet, dress, bath/shower, get in/out of bed or chairs, and walk without difficulty | Can use the telephone, manage money, prepare meals, do light housework, shop for personal items, and do heavy housework without difficulty |
| Stage 1 : mild disability | Eating, toileting, dressing, and bathing/showring are no difficult ; may have difficulty getting in/out of bed or chairs and/ or walking | Using the telephone, managing money, preparing meals,and doing light housework are not difficult ;may have difficulty shopping for personal items and /or doing heavy housework |
| Stage 2 :moderate disability | Eating and toileting are nit difficult ; may have difficulty dressing , bathing/showering,getting in/out of bed or chairs,and §or walking | Using the telephone and managing money are not difficut ; may have difficulty preparing meals, doing light housework ; shopping for personal items and /or doing heavy housework |
| Stage 3 : severe disability | Difficulty with eating and/or toileting but not with all ADLs | Has difficulty using the telephone and/or managing money but not all IADLs are difficult |
| Stage 4 : complete disability | All ADLs are difficult | all IADLs are difficult |
