# Supplementary material for: Protocol for a qualiquantitative study of accessibility of sexual and reproductive health services among women with motor disabilities in Morocco
Source: PLoS One. 2024 Oct 29;19(10):e0311608. doi: 10.1371/journal.pone.0311608 (PMC11521270; doi:10.1371/journal.pone.0311608)
Supplement: Attachment — Submitted filename: Response-To-Reviewers.docx [file pone.0311608.s002.docx]

Reviewer #1

Thank you for your insightful feedback on my manuscript. I appreciate the time and effort you put into reviewing it and providing constructive suggestions. Below, I address your comments point by point:

Comment : Abstract length

Response : I have reduced the abstract by approximately 150 words, ensuring it remains concise while retaining key information for both readers and search engines.

Comment : Research question

Response : I have refined the research question to make it clearer and easier to identify, providing a precise summary of the research objective at the beginning of the abstract. The primary research question is now explicitly stated, followed by the secondary questions.

Comment :Materials and Methods section

Response : I have restructured this section based on your suggestions, detailing the process in several stages. This includes:

- Study characteristics: A comprehensive overview of the study design, including variables measured, methodologies, and the equipment used.
- Research adaptations: I have added details on any adjustments made to the research protocol and highlighted any unforeseen events that occurred. These revisions ensure the study is reproducible by other researchers.

Comment : Discussion section expansion

Response : Following your recommendation, I have expanded the discussion to elaborate on the expected outcomes, potential limitations, and future research directions.

Once again, thank you for your valuable feedback. I believe these revisions have strengthened the manuscript and look forward to your further comments.

Reviewer #2

Thank you for your insightful feedback on my manuscript. I appreciate the time and effort you put into reviewing it and providing constructive suggestions and sincerely appreciate your thoughtful review. Below, I address each of the points you raised:

Comment : Relevance and Importance

Response : Thank you for acknowledging the importance of the study on access to sexual and reproductive health (SRH) services for women with physical disabilities in Morocco. Given the lack of research in non-Western contexts, we aim to fill this gap and contribute to global efforts in ensuring equitable access to SRH services, particularly for marginalized populations like women with disabilities.

Comment : Clear Objectives

Response : We structured the abstract to clearly outline the study's objective, which is to identify the barriers and facilitators women with disabilities encounter when seeking SRH services. Our goal is to produce findings that are both informative and actionable, with the potential to influence policy and practice.

Comment : Methodological Rigor

Response : We appreciate your recognition of the strength in our mixed-methods approach. By integrating quantitative and qualitative data, we aim to provide a comprehensive understanding of the issue. The combination of statistical data and personal narratives offers a deep exploration of the challenges faced by these women, along with healthcare professionals' perspectives on how these can be addressed.

Comment : Innovative Aspects

Response : We are encouraged by your acknowledgment of our recruitment strategy, which includes collaboration with NGOs, local organizations, and the use of social media to reach a diverse and inclusive participant pool. This approach will enable us to capture experiences from both urban and rural settings.

Comment : Potential Impact

Response : We are grateful for your recognition of the study's potential to influence policy both locally and globally. Our goal is to use empirical data to advocate for more inclusive SRH services and to provide recommendations that reflect the lived experiences of women with disabilities.

Comment : Challenges and Limitations

Response : The reviewer’s observation regarding potential challenges in reaching rural populations and the reliance on self-identification for inclusion criteria is well-founded. We acknowledge that these are limitations we have considered. In response, we will made efforts to ensure robust recruitment strategies in rural areas, working closely with local organizations familiar with these communities. Additionally, we aim to mitigate bias by employing flexible inclusion criteria to include with diagnosed physical impairments affecting mobility or walking, as defined by the Centers for Disease Control and Prevention and ensuring that our sampling captures a wide range of experiences

Comment 7 :Ethical Considerations

Response :We have carefully considered your point about ethical concerns. A verbal informed consent process will be implemented to accommodate participants with varying literacy levels, ensuring their full understanding of the study. Participants with significant cognitive impairments will be excluded to maintain the ethical integrity of the study, though we recognize the need for future research that includes such individuals in an ethical and inclusive manner.

Comment : Generalizability

Response : While our findings are specific to Morocco, we agree that themes of accessibility and inclusion are broadly applicable across various cultural contexts. We hope that our research will inspire further studies in other low- and middle-income countries.

Comment : Future Directions

Response :We appreciate your acknowledgment of the study's potential for future research. We envision this work as a foundation for longitudinal studies to assess the impact of our recommendations over time, focusing on the evolution of SRH services in response to our findings.

Comment : Contribution to Global Health

Response : It is gratifying to see that our study aligns with global health priorities, particularly the Sustainable Development Goals (SDGs). By addressing the intersection of disability and SRH, we hope to contribute to the ongoing discourse on health equity and inclusive health systems.

Once again, thank you for your valuable feedback. I believe these revisions have strengthened the manuscript and look forward to your further comments.
